# Toilet Training Readiness Scale for 0–5-Year-Old Children: A New Measurement Tool Based on a Child-Centred Approach

**DOI:** 10.3390/children11091149

**Published:** 2024-09-23

**Authors:** Adnan Barutçu, Burak Mete, Hakan Demirhindi, Saliha Barutçu, Aliye Kıdı, Nurdan Evliyaoğlu

**Affiliations:** 1 Department of Paediatrics, Faculty of Medicine, Cukurova University, Adana 01330, Türkiye; abarutcu@cu.edu.tr (A.B.); akidi@cu.edu.tr (A.K.); enurdan@cu.edu.tr (N.E.); 2 Department of Public Health, Faculty of Medicine, Cukurova University, Adana 01330, Türkiye; bmete@cu.edu.tr; 3 Cukurova District Health Directorate, Adana 01310, Türkiye; saliha.barutcu@saglik.gov.tr

**Keywords:** toilet training, toilet learning, child, healthy child follow-up

## Abstract

Background and Objectives: There is no standardised approach to toilet training in children. This study aimed to determine the factors affecting the duration of toilet training in children aged 0–5 years and to develop a tool to assess the child’s readiness to start toilet training. Materials and Methods: This cross-sectional study was conducted on 409 children aged 0–5 years. Social, economic, behavioural, and developmental characteristics that are effective in toilet training in healthy children were evaluated. A scale assessing children’s readiness for toilet training (Toilet Training Readiness Scale-TTRS) was developed and content validated. Results: The mean age of the 409 children included in this study was 44.69 ± 13.07 months (min = 4; max = 60 months). The mean age of initiation of toilet training was 26.8 months. Most frequently, urine and faeces trainings were started together (52.1%). In the logistic regression analysis performed to evaluate the factors affecting the duration of toilet training, it was found that the TTRS score, mother’s employment status, family type, child’s first reaction, toilet type, and continuity of training were important predictors. The duration of toilet training showed a weak negative correlation with the scores obtained from the TTRS and the number of children in the family but a weak positive correlation with the age at the beginning of toilet training. The TTRS scores were inversely proportional to the duration of toilet training. Conclusions: Family characteristics, socioeconomic conditions, and readiness of the child for and no interruption in toilet training are important in completing toilet training in a short time and successfully. If a child-focused approach is adopted, evaluating the child from this point of view and initiating the training at the appropriate time may help to complete a more successful and shorter toilet training. We recommend that the scale we have developed be studied in other studies and different groups.

## 1. Introduction

Toilet training is defined as the child being aware of the need to pass urine and faeces and being able to initiate the action without being reminded or prepared by the parents [1,2]. Generally, starting before 2 years (24 months) of age is not recommended [1].

The preparation skills and physical development the child requires to undertake successful toileting, usually occur between 18 months and 2.5 years of age. Current acceptance worldwide is that toilet training should be introduced to children between the ages of 2 and 3 years [1]. The American Academy of Paediatrics and the Canadian Paediatric Society report that the most physiologically, cognitively, and behaviourally appropriate time to start toilet training is between 18 and 24 months [3,4]. In children with typical development, the skills of recognising people, imitation, controlling simple stimuli and developing autonomy are completed between the ages of 2 and 3 years. Whether the children are ready for toilet training can be deducted from their cognitive and psychological reactions to their parents [5,6]. The readiness can be evaluated via physical, cognitive, and emotional aspects. Physical signs encompass a child’s physiological and motor abilities like control of sphincters and ability to perform gross motor skills such as walking, sitting, and squatting comfortably. Cognitive development accelerates as children begin to explore the outside world and themselves, these cognitive signs include understanding what is said, following simple instructions, and being able to say words related to the bathroom (toilet, sink, soap, toilet paper, etc.). Emotional aspects are signs such as the child being uncomfortable when his/her nappy is dirty, asking for it to be changed or trying to take it off himself/herself, wanting to be liked by the parents, striving to get reward or praise, turning towards the toilet or bathroom when he/she needs to use the toilet and wanting to use it [5,6,7]. Studies on toilet training in children are limited in the literature. However, many parents need guidance from their physicians on how best to fulfil this training. Psychological theories, paediatric recommendations, and parental practices related to toilet training have changed considerably in the last century [8]. In the last 50 years, the ages of initiation and completion of toilet training for children with normal neuropsychomotor development have been delayed from 18 months to 24–36 months and from 24 months to 36–39 months, respectively [9,10]

At the beginning of the twentieth century, toilet training, which was expected to develop naturally in line with the mother’s instincts, underwent a sharp transformation with the development of the behaviourist approach [11]. Two main classifications of toilet training have been used: the child-focused approach and the structured behavioural approach. Most children were trained with the structured behavioural approach starting early, but by the time toilet training was completed, they were similar in age to those using the child-focused approach. In the few reported studies, success rates were better with the child-focused approach. The lowest success rate was reported with the daytime dampness alarm approach. There is no consensus on the best method to use, as this depends on parental preferences, expectations, and cultural differences [12].

The duration of toilet training is affected by multiple factors which can be grouped as follows:

(a) Personal factors: sex and age of the child or the age and educational status of the parents and the psychological status of the child;
(b) Socio-demographic factors: the family and environment where the child lives, the place of residence, and the socioeconomic status of the family;
(c) Educators/caregivers: family or non-family members at home or nursery school; how much information the educator has about toilet training; and the attitude of the parents during toilet training;
(d) Methods and tools: the style of the toilet, i.e., Western (also named as sitting or flush toilet) versus Oriental (also known as squat); location of the toilet: inside or outside the house; and the methods and tools used in toilet training.

Health conditions such as chronic diseases may render the process more difficult [13,14,15].

In this study, it was aimed to determine the cognitive, behavioural, and sociocultural factors affecting the duration of toilet training in children aged 0–5 years who applied to the social paediatrics outpatient clinic in a university hospital and to develop a scale, i.e., a form that will assess the child’s readiness to start toilet training that can help clinicians during the counselling process given to families.

## 2. Materials and Methods

### 2.1. Study Type, Place, and Time

This cross-sectional study conducted between April 2023 and April 2024 included the mothers of children between 0 and 60 months of age who had completed toilet training with their children or whose toilet training was still ongoing and who applied to the child health follow-up outpatient clinic of the Department of Social Paediatrics at the Faculty of Medicine of Çukurova University in Adana, Turkey.

### 2.2. Sampling

The sample size analysis with d = 0.1, 1 − β = 0.95, and α = 0.01 revealed the minimum sample size to be reached in the study as 391. Participants were reached using a convenience sampling method. The questionnaire form was filled face-to-face, and the evaluation of each participant took an average of 30 min. This study was completed with 409 participants.

-Inclusion criteria: parents of children born at term and children who did not have any chronic diseases were included in this study after having signed a written consent form;

-Exclusion criteria: children with a history of premature birth, physical or mental disability, and anomalies that might affect bladder function and anal emptying were excluded from this study.

### 2.3. Ethics

Ethical approval was obtained from the Non-interventional Clinical Research Ethics Committee of the Faculty of Medicine at Çukurova University (decree no: 16, meeting no: 111, dated 21 May 2021).

### 2.4. Questionnaire Form

The form consisted of questions regarding the sociodemographic characteristics of mothers and children, in addition to the scale designed to determine the signs of children’s readiness for toilet training and the attitudes and behaviours of mothers towards this training.

Although there is no consensus on when a child is considered fully trained, it is defined as when he/she is aware of his/her own need to eliminate urine and stool and initiates the act without being remembered or prepared by parents or caregivers. In our study, while questioning the completion of toilet training, we took into consideration whether the child spent the night dry, whether the child went to the toilet by controlling faeces and urine during the day and whether the child was completely weaned from the nappy [2].

### 2.5. Toilet Training Readiness Scale (TTRS)

The scale we have developed consists of 10 content-validated questions that aim to evaluate children’s readiness for toilet training physiologically, cognitively, and psychologically [5].

#### Content Validity

- Creating an Item Pool:

A questionnaire consisting of 15 questions evaluating the developmental characteristics of a child was created by the researchers based on their clinical observations and the literature to evaluate the readiness of children for toilet training [16,17]. The items were expressed as positive or negative, and the scale items were expressed in understandable language. Care was taken not to have more than one meaning in an item. The answers to the statements used in the scale consisted of “yes” or “no”.

- Content Validity (Expert Opinion)

Validity is a concept related to the extent to which the instrument (which is a scale in our study) accurately measures the desired characteristic in question. The content validity is the indicator of whether the items constituting the instrument are sufficient in terms of quantity and quality to measure the behaviour in question. One of the frequently used methods to test content validity is seeking expert opinions [18]. In our study, the 15-item draft scale was sent to five faculty member paediatricians. How this number was calculated is explained below.

- Calculation of Content Validity Ratio (CVR) and Content Validity Index (CVI)

The content validity ratio (CVR) is an internationally recognised item statistic to determine content validity; in other terms, it is used to decide whether to include or reject an individual item in any measurement tool [15]. It is calculated with the following formula:

$$CVR=\frac{Nu}{N/2}-1$$

where Nu indicates the number of experts who evaluated the item as “essential”, and N indicates the total number of experts who expressed any opinion (“essential”, “useful, but not essential”, or “not necessary” on this item. The CVR has a value between −1 (perfect disagreement) and +1 (perfect agreement). If all participants rate any item in the scale as “essential”, the CVR value of that item becomes 1. If the CVR takes a 0 (zero) or negative (less than zero) value, it means that the item with such a value does not have content validity, and all such items in the scale are promptly eliminated [19,20,21]. Ayre and Scally (2014) pointed out that if the number of experts participating in the study increased or decreased even by one person, the critical values of the CVRs would change and proposed a table to determine the critical (minimum acceptable) CVR (CVR_critical_) by using different statistical analyses with α (Type I error probability) set to 0.05. To summarise, this table gave the minimum number of experts required to agree that an item was essential and thus which items should be included or discarded from the final instrument could be determined [20].

When the hypotheses proposed by Lawshe [21], Wilson et al. [19], and Ayre and Scally [20] were evaluated together, the common consensus was to use the aforementioned table of Ayre and Scally, where CVR_critical_ was reported as 1.00 when the panel included five experts and α = 0.05 is the significance level [21]. In our study, 5 items were removed because they had zero or negative values with a resulting total of 10 items being accepted in terms of content validity in the final version of our scale. The content validity index (CVI) is the mean of CVRs of all the items included in the tool [22]. Assuming that the draft scale used in our study had a single dimension, the CVI value for 10 items was calculated as 1 for a single dimension. Since CVI = 1, the scale could be assessed as valid in terms of content. The questions of the scale are given in Table 1. After the content was validated, the scale was administered to the families. Parents were asked to answer “yes” or “no” to each question. The answer “yes” was scored as “1 point” while “no” was scored as “0 points”. Thus, the total score on the scale ranged between 0 and 10. Increased scores were interpreted in favour of more likely readiness for toilet training. The reliability analysis revealed a Cronbach alpha value of 0.567.

### 2.6. Statistical Analyses

SPSS 20.0 (IBM Corp., Armonk, NY, USA) and JAMOVI 2.3 [23] software were used for data analysis. The chi-square test, binary logistic regression, multinominal logistic regression, mediation, Spearman correlation, and ROC analyses were used. A *p* < 0.05 value was considered statistically significant.

## 3. Findings

The mean age of the 409 children included in our study was 44.69 ± 13.07 months (min = 4; max = 60). The mean age for toilet training initiation was 26.8 months. Females constituted 50.6% of the children. Sociodemographic data of the children and their families are given in Table 2.

A total of 66.7% of the mothers started toilet training because they thought that their child was ready. Most frequently, trainings were started together for both urine and faeces (52.1%). The duration of training was 2–7 days for 24.4% of the children and 8–15 days for 27.9%. Most of the children were enthusiastic on the first attempt (64.1%). The most preferred toilet type was the Oriental (also known as squat) style. Other information about the toilet training process of the families is given in Table 3.

A significant logistic regression model (Forward LR method) was constructed (omnibus test *p* < 0.001). The explanatory power of the model was 27.3%, and the accuracy rate was 72.8%. Among the variables included in the model, TTRS score, mother’s employment status, family type, child’s first reaction, toilet type, and continuity of training were found to be significant. The probability of completion of toilet training in less than 15 days was found to be 2.69-times higher in children with mothers who were employed, 2.09-times higher for those who lived in nuclear families, 2.79-times higher for children whose first reaction was enthusiastic, 6.89-times higher for those who used a Western-style toilet, 3.68-times higher for those trained in the Oriental style, 3.86-times higher for potty users, and 4.22-times higher for those whose toilet training was not interrupted. Each one-unit increase in the TTRS score increased by 1.30 times the probability of the duration of toilet training being shorter than 2 weeks (Table 4).

**Figure 1:**
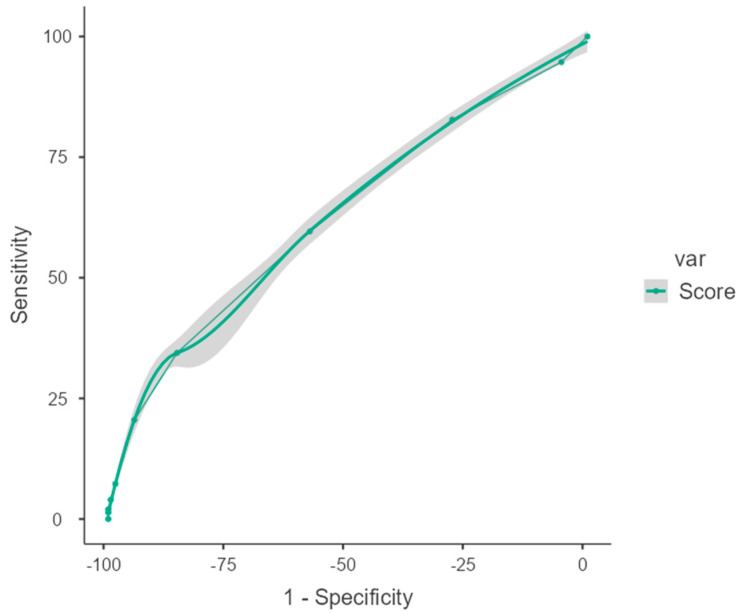
ROC analysis of Toilet Training Readiness Scale scores (area under the curve).

When the correlations between the TTRS score and duration of toilet training, mother’s age, number of children, and age at the beginning of toilet training were examined, TTRS scores were found to show a significant weak correlation in the negative direction with the duration of toilet training or with the number of children but a significant weak correlation in the positive direction with the age at the beginning of toilet training. There was also a weak positive correlation between the number of children and the duration of toilet training (Table 5). The optimum cut-off value for the TTRS developed in our study was found to be 6 (Table 6, Figure 1).

The scores obtained from the Toilet Training Readiness Scale were classified according to the optimum cut-off value that we found in our study, and their effect on the duration of toilet training was evaluated. It was found that the probability of completing toilet training in a period longer than 1 month increased by 5.43 times in children who had a score below 6 (Table 7).

## 4. Discussion

Although toilet training is a challenging experience for parents and children, it constitutes one of the most important developmental stages of childhood. The initiation and completion of toilet training is under the influence of many facilitating and inhibiting factors [24]. In this study, the factors that are effective in toilet training in children aged 0–5 years were evaluated, and a scale was developed to assess the child’s readiness for toilet training (TTRS; Toilet Training Readiness Scale). In our study, the mean age at toilet training initiation was 26.8 months. The important predictors of the duration of toilet training in children were TTRS score, mother’s employment status, family type, child’s first reaction, toilet type, and continuity of training. There was a significant weak negative correlation between the TTRS score and the duration of toilet training, while there was a significant weak positive correlation with the age at toilet training initiation.

In the study conducted by Koç et al., the mean age of initiation of toilet training was 22.05 ± 6.73 months. The completion age was later in infants whose mothers had 12 years or more of education compared to the others, and the earliest completion age was observed in families using the punishment method. The duration of education was found to be longer in families living in rural and semi-urban settlements, in mothers with less than 5 years of education, in unemployed mothers, in children living in houses without indoor bathrooms, and in families using reusable washable nappies [14]. In the study conducted by Netto et al., it was found that prematurity and mothers working away from the home delayed toilet training [25]. In our study, it was found that the working status of the mother was significantly impactful, and the duration of toilet training was shorter in working mothers. This may be related to the social change over time. It may be related to working women being more conscious or sharing experiences and exchanging ideas with other mothers on these issues and being aware of correct practices. The fact that working mothers spend less but more productive time with their children than non-working mothers may also be one of the explanatory factors of this situation. In the study conducted by Önen et al., the duration of toilet training in children under five years of age was found to be lower in families with low-income levels and children living in nuclear families. The duration of toilet training was found to be longer in those who started toilet training before 18 months of age or later than 30 months, in those living in slums, and in those who experienced an event affecting their psychology compared to other groups [26]. Mrad et al. examined the effect of the child-focused toilet training method approach in children with Down syndrome and children with normal psychomotor development and found that the duration of toilet training was longer in children with Down syndrome [27]. In a cohort study conducted by Mota et al. on a total of 3281 children born in Brazil, it was found that mothers with a longer education period and from higher social class weaned their children from nappies later. The probability of nappy weaning increased when the number of children living at home was higher (RR = 1.32) and the child could communicate the need to go to the toilet to others (RR = 11.74) [28]. In the study conducted by Kural and Köse, it was found that the ages of children who encountered problems in toilet training were higher than those without problems. The rate of problems in toilet training in boys was found to be statistically significantly higher than that in girls. The rate of problems in toilet training of preterm children was higher than that of term children. Refusal of toilet training was found to be significantly higher in children with developmental language and speech delays [29]. The common finding of our study and these studies was the relationship between child development and toilet training success. There were no consistent findings between socioeconomic factors and toilet training, and the period in which the studies were conducted and the social characteristics might explain this. However, it is noteworthy that there was a close relationship between the developmental characteristics of the child and the duration and success of toilet training.

In the study conducted by Blum et al., advanced age at the beginning of toilet training, refusal to defecate and constipation were found to be predictors of the tendency to complete toilet training at a later age [30]. Vermendal et al. compared gradual child-focused education and structured endpoint-focused toilet training in healthy children. It was emphasised that there was no consensus on the most appropriate age to start toilet training or the expected average age to complete the training in the literature reviewed, where studies focusing on this subject were very few. Recent studies showed that children completed toilet training much later than previous generations. It was reported that the child started training between 24 and 36 months, and the current trend was completed later than previous generations [31]. In the study conducted by Kaerts et al., signs of readiness for toilet training were analysed, and 21 signs of readiness were identified: the child 1. could imitate behaviour, 2. could sit steadily and without assistance, 3. could walk without assistance, 4. could pick up small objects, 5. could say “no” as a sign of independence, 6. had voluntary control over bowel and bladder reflex actions, 7. could understand and respond to instructions, questions, or explanations and fulfil simple commands, 8. could express the need to evacuate through non-verbal communication (imitation, posture, or gestures, such as going to the toilet or grabbing the potty) or words, 9. could often indicate his/her trousers to be wet/dirty, 10. enjoyed putting things in containers, 11. had awareness of bladder sensations and the need to urinate, 12. understood toilet-related words and had a sufficient vocabulary of his/her own, 13. wanted to participate in toilet training, cooperated and showed interest in toilet training, 14. had a larger bladder capacity, 15. insisted on completing tasks without help and was proud of new skills, 16. wanted the potty to be clean and was uncomfortable with wet or dirty nappies, 17. wanted to wear adult clothes, 18. could pull his/her clothes up and down, 19. stayed overnight without bowel movements, 20. began to put things back where they belonged, and 21. the child could sit still on the potty for 5–10 min. The researchers emphasised that there was no consensus on which or how many signs of readiness to use. According to the signs of readiness, the moment of initiating toilet training can vary greatly. More studies were needed to define which readiness signs were the most important and how they could be easily detected [6].

Although there are various methods of toilet training, it is not clear when the right time to start training is, and this varies from child to child. There are many signs indicating that children are ready for toilet training. However, there is no consensus on any of them, and there is no clear attitude on deciding which one is the most important. Based on this, in our study, a child-centred scale was developed to objectively determine the readiness of children for toilet training where multiple signs were evaluated. The increase in the scores obtained from this scale was found to be associated with the shortness of toilet training. Scores of 6 and above indicated that the child was ready to start and successfully complete toilet training. It was found that the probability of completing toilet training in a longer period of time (more than one month) increased by 5.43 times in children who scored below 6 on the scale. In addition, the 10th item on the scale, “My child feels discomfort after defecating in his/her nappy/diaper” was found to be the most important predictor of toilet training success. Preparation for initiation of toilet training depends on the individual situation of the child [16]. There is no right age for giving toilet training to a child; the scale developed in our study may help clinicians to objectively evaluate whether healthy children aged 0–5 years are ready for toilet training.

Toilet training is a universal challenge for children, parents, and doctors. Although parents and physicians have addressed this issue for generations, there is still no consensus on the best method or even a standardised definition of toilet training. This uncertainty is partly due to the wide range of parental preferences and expectations regarding toilet training. In addition, few studies compared the effectiveness of different strategies, which limited the ability of physicians to make evidence-based recommendations. For these reasons, toilet training remains a complex behavioural and developmental stage with a variety of acceptable approaches and methods [32]. Many toilet training methods are available. The American Academy of Paediatrics recommends a child-centred approach for toilet training, which means that it should be started when the child is physically and psychologically ready [5,10,33], which is also the main approach in our study.

In the follow-up of healthy children, caregivers of children under 5 years of age should be informed about the importance of toilet training, clues indicating readiness for toilet training, and positive approaches/methods. The results of our study allow these evaluations to be made more objectively. There is no consensus on the criteria for preparation for and success of toilet training. Our study will guide the literature and parents and health professionals in terms of evaluating the readiness for toilet training in children under 5 years of age.

Although the readiness of healthy children for toilet training was evaluated from a neuropsychomotor perspective in our study, sometimes a delay in toilet training may be a harbinger of abnormal conditions. Further consideration of parent–child communication, children’s development and level of toilet skills may contribute to the development of toilet training approaches that better meet the needs of families of children with disorders such as autism spectrum disorder [34,35].

## 5. Conclusions

Our results show that family characteristics, socioeconomic and cultural conditions, signs of readiness in the child’s physical and mental development, and the absence of interruption in toilet training are important in completing toilet training shortly and successfully. If a child-oriented approach is adopted, evaluating the child in this respect and starting education at the appropriate time may help to complete a more successful and shorter toilet training. A score of 6 and above obtained on the scale we developed indicates that the child is physically and mentally ready for toilet training. Children who score below 6 on the scale are 5.4 times more likely to complete toilet training in more than 1 month. We recommend that the validity of the scale we developed—Toilet Training Readiness Scale—be investigated in clinics in different cultures and societies also including children with physical or mental development problems and that our results be compared with other studies.

## Figures and Tables

**Table 1:** Toilet Training Readiness Scale questions.

	Yes	No
My child may imitate other people’s behaviour or actions		
My child can stand or walk unaided		
My child has enough language skills to explain that he/she needs to go to the toilet		
My child can say “no” as a sign of independence		
My child can understand and follow up simple commands		
My child can pick up things on his/her own and put them back where they belong		
My child has a tendency to and interested in using the toilet		
My child gives a favourite object to a favourite person		
My child can dress or undress on his/her own		
My child feels uncomfortable after wetting/pooping him/herself or his/her nappy		

**Table 2:** Characteristics of children and families.

Features	Mean ± S.D.	Median (Min–Max)
Age of mother (years)	33.14 ± 6.07	33 (21–54)
Age of the child (months)	44.69 ± 13.07	48 (4–60)
Number of children in the family	2.22 ± 0.98	2 (1–7)
Age at toilet training initiation (months)	26.86 ± 7.74	24 (2–54)
		**n (%)**
Sex	Female	207 (50.6)
	Male	200 (48.9)
Educational diploma of the mother	Illiterate	18 (4.4)
	Literate	18 (4.4)
	Primary education	84 (20.5)
	Secondary Education	137 (33.5)
	Higher Education	151 (36.9)
Employment status of the mother	Working	166 (40.6)
	Not working	239 (58.4)
Monthly income of the household	Under the minimum wage	58 (14.2)
	Equal or above the minimum wage	347 (84.8)
Social security	Present	363 (88.8)
	Absent	43 (10.5)
Place of residence	Province	309 (75.6)
	District	74 (18.1)
	Village	21 (5.1)
Family type	Nuclear	349 (85.3)
	Extended	54 (13.2)

**Table 3:** Distribution of characteristics related to toilet training.

		n	%
Which reason was most influential in your decision to start toilet training?	I started because I wanted to	104	25.4
	I started because I thought my child was ready	273	66.7
	I started at the suggestion/request of relatives	23	5.6
	Influence of press and media organs	4	1.0
Which one did you start your child’s toilet training with?	Urine (pee)	178	43.5
	Faeces (poop)	18	4.4
	Both of them	213	52.1
How long did your child’s toilet training last?	2–7 days	100	24.4
	8–15 days	114	27.9
	16–30 days	80	19.6
	31 days and longer	74	18.1
	I don’t remember	17	4.2
	Still continues	24	5.9
How often did you take your child to the toilet during toilet training?	Every hour on the hour	130	31.8
	Every 2 h	97	23.7
	According to the needs of the child	182	44.5
How long did you keep your child in the toilet during toilet training?	Shorter than 5 min	188	46.0
	5–10 min	124	30.3
	Longer than 10 min	38	9.3
	Until he/she finished	58	14.2
How did your child react after the first attempt at toilet training?	He/she was enthusiastic and strived for it	262	64.1
	He/she was not very enthusiastic and doing it through my efforts	115	28.1
	He/she indicated by his/her actions, behaviour and/or verbally that he/she did not want to do this at that moment	29	7.1
Which type of toilet did you use for the toilet training of your child?	Western style (sitting or flush toilet)	113	27.6
	Oriental style (also known as squat)	138	33.7
	Toilet pot	126	30.8
	Adapter over normal adult toilet	32	7.8
Did you restrict your child from drinking liquids after you started toilet training?	Yes	65	15.9
	No	344	84.1
Has your child’s toilet training been interrupted for any reason?	Yes	57	13.9
	No	351	85.8
Did you have any difficulties during your child’s toilet training?	Stubbornness	58	14.2
	Fear of the toilet	81	19.8
	Constipation	41	10.0
	Other (Specify)	18	4.4
	I didn’t have any difficulties	223	54.5
Did you use punishment when toilet training your child?	Hurting him/her slightly	9	2.2
	Keeping at the toilet until he/she peeped/pooped	37	9.0
	Scaring	10	2.4
	Reprimanding	19	4.6
	Other	7	1.7
	No punishment	327	80.0
Have you ever felt that you put pressure on your child during toilet training?	Sulking and not talking	26	6.4
	Physical violence	7	1.7
	Depriving him/her of his/her favourite food	4	1.0
	Depriving him/her of his/her favourite game/toy	18	4.4
	Other	11	2.7
	No pressure	341	83.4
Did you use a reward method when toilet training your child?	Caressing/praising	186	45.5
	Giving a favourite food	94	23.0
	Buying toys	86	21.0
	Other	6	1.5
	No reward	114	27.9
How often did you put nappies under your child during toilet training?	A nappy was always tied	35	8.6
	A nappy was tied only at night	207	50.6
	No nappy day and night	141	34.5
	Other	26	6.4
Did you use facilitating methods when toilet training your child?	Baby doll wetting the bed	31	7.6
	Toilet training themed book reading	51	12.5
	Singing/listening a song	100	24.4
	Toilet equipment that will interest him/her	43	10.5
	Other	11	2.7
	No method was used	210	51.3
Did you consider the seasonal conditions when you started toilet training your child?	Yes	100	24.4
	No	307	75.1
Where does your child go to the toilet after toilet training?	Toilet	347	84.8
	The potty, in his/her own room	20	4.9
	The potty, in any corner of the house	19	4.6
	Other	17	4.2
Was there anyone who supported your child during toilet training?	No support	182	44.5
	My mate	141	34.5
	My carer	22	5.4
	My relatives	59	14.4
	Other	22	5.4
Which of sources of information did you use about toilet training for your child?	Relatives	113	27.6
	Neighbours	39	9.5
	Books	71	17.4
	Media	29	7.1
	Internet	120	29.3
	Other	11	2.7
	No information received	150	36.7
Have you received any information from a health institution about your child’s toilet training?	Yes	30	7.3
	No	375	91.7

**Table 4:** Logistic regression analysis for prediction of toilet training duration *.

	B	*p*	O.R.	95% C.I. for O.R.	
				Lower	Upper
TTRS score	0.269	0.002	1.30	1.104	1.552
Mother’s employment status (ref: Not working)	0.993	<0.001	2.69	1.530	4.763
Family type (ref: Extended family)	0.737	0.044	2.09	1.020	4.283
How did your child react after the first attempt at toilet training? (ref: He/She did not want to do)					
Enthusiastic and strived for it	1.026	0.038	2.79	1.057	7.360
Not enthusiastic but with the effort of the mother	0.163	0.752	1.17	0.429	3.230
Which toilet did you use when toilet training your child? (ref: Adapter over normal adult toilet)					
Western style	1.931	<0.001	6.89	2.378	20.003
Oriental style	1.305	0.015	3.68	1.288	10.561
Toilet pot	1.352	0.009	3.86	1.400	10.673
Has your child’s toilet training been interrupted for any reason? (ref: Yes)	1.441	<0.001	4.22	1.895	9.414
Constant	−2.926	<0.001			

* Participants with ongoing toilet training were excluded. Variables entered in the forward LR method: TTRS score, mother’s educational status, mother’s employment status, family type, age at toilet training initiation, child’s age, number of children, child’s reaction after the first toilet training attempt, toilet type, interruption of toilet training, forcing during toilet training, punishment during toilet training, applying pressure on the child, reward method, using facilitative methods, and applying traditional methods.

**Table 5:** Various correlations with Toilet Training Readiness Scale scores *.

		Scheme	Toilet Training Duration	Age of Mother	Number of Children in the Family	Child’s Age	Age at Toilet Training Initiation
**Score**	r	1.000	−0.239	0.019	−0.155	0.074	0.202
	*p*		**0.000**	0.364	**0.002**	0.088	**0.000**
**Toilet training duration**	r		1.000	0.000	0.108	0.012	0.051
	*p*			0.497	**0.023**	0.411	0.169
**Age of mother**	r			1.000	0.145	0.163	0.039
	*p*				**0.004**	**0.001**	0.243
**Number of children**	r				1.000	0.122	−0.094
	*p*					**0.012**	**0.041**
**Child’s age**	r					1.000	0.150
	*p*						**0.003**
**Age at toilet training initiation**	r						1.000
	*p*						

* Participants with ongoing toilet training were excluded. Bold values indicate significant *p* values.

**Table 6:** Optimum cut-off values of Toilet Training Readiness Scale scores.

Cut-Off	Sensitivity (%)	Specificity (%)	PPV (%)	NPV (%)	Youden’s Index	AUC	Metric Score
0	1.32	100.00	100.00	57.55	0.01325	0.628	1.01
1	1.99	100.00	100.00	57.70	0.01987	0.628	1.02
3	3.97	99.50	85.71	58.09	0.03478	0.628	1.03
4	7.28	98.51	78.57	58.70	0.05800	0.628	1.06
5	20.53	94.55	73.81	61.41	0.15084	0.628	1.15
**6**	**34.44**	**85.64**	**64.20**	**63.60**	**0.20081**	**0.628**	**1.20**
7	59.60	57.92	51.43	65.73	0.17523	0.628	1.18
8	82.78	28.22	46.30	68.67	0.10999	0.628	1.11
9	94.70	5.45	42.81	57.89	0.00148	0.628	1.00
10	100.00	0	42.78	N/A	0.00000	0.628	1.00

Bold line indicates the optimum cut-off value; PPV, Positive Predictive Value; NPV, Negative Predictive Value; AUC, Area under the curve.

**Table 7:** Multinomial logistic regression analysis predicting the duration of toilet training.

Toilet Training Time		B	*p*	O.R.	95% Confidence Interval for O.R.	
					Lower Bound	Upper Bound
8–15 days	Intercept	0.157	0.278			
	[score < 6]	−0.340	0.585	0.712	0.210	2.413
	[score ≥ 6]	0				
16–30 days	Intercept	−0.288	0.077			
	[score < 6]	0.894	0.094	2.444	0.860	6.948
	[score ≥ 6]	0				
31 days and longer	Intercept	−0.488	0.005			
	[score < 6]	1.692	0.001	5.432	2.053	14.375
	[score ≥ 6]	0				

The reference category is 2–7 days; participants with ongoing toilet training were excluded.

## Data Availability

The data that support the findings of this study are available on request from the corresponding author. The data are not publicly available due to privacy or ethical restrictions.
